# Connected speech as a marker of disease progression in autopsy-proven Alzheimer’s disease

**DOI:** 10.1093/brain/awt269

**Published:** 2013-10-18

**Authors:** Samrah Ahmed, Anne-Marie F. Haigh, Celeste A. de Jager, Peter Garrard

**Affiliations:** 1 Stroke and Dementia Research Centre, St George’s, University of London, Cranmer Terrace, London, SW17 0RE UK; 2 Nuffield Department of Clinical Neurosciences, University of Oxford, John Radcliffe Hospital, OX3 9DU UK; 3 Oxford Project to Investigate Memory and Ageing (OPTIMA), Nuffield Department of Medicine, University of Oxford, John Radcliffe Hospital, Oxford, OX3 9DU UK; 4 School of Public Health & Family Medicine, Faculty of Health Sciences, University of Cape Town, South Africa

**Keywords:** Alzheimer’s disease, aphasia, neuropsychological tests, language, connected speech analysis

## Abstract

Although an insidious history of episodic memory difficulty is a typical presenting symptom of Alzheimer’s disease, detailed neuropsychological profiling frequently demonstrates deficits in other cognitive domains, including language. Previous studies from our group have shown that language changes may be reflected in connected speech production in the earliest stages of typical Alzheimer’s disease. The aim of the present study was to identify features of connected speech that could be used to examine longitudinal profiles of impairment in Alzheimer’s disease. Samples of connected speech were obtained from 15 former participants in a longitudinal cohort study of ageing and dementia, in whom Alzheimer’s disease was diagnosed during life and confirmed at post-mortem. All patients met clinical and neuropsychological criteria for mild cognitive impairment between 6 and 18 months before converting to a status of probable Alzheimer’s disease. In a subset of these patients neuropsychological data were available, both at the point of conversion to Alzheimer’s disease, and after disease severity had progressed from the mild to moderate stage. Connected speech samples from these patients were examined at later disease stages. Spoken language samples were obtained using the Cookie Theft picture description task. Samples were analysed using measures of syntactic complexity, lexical content, speech production, fluency and semantic content. Individual case analysis revealed that subtle changes in language were evident during the prodromal stages of Alzheimer’s disease, with two-thirds of patients with mild cognitive impairment showing significant but heterogeneous changes in connected speech. However, impairments at the mild cognitive impairment stage did not necessarily entail deficits at mild or moderate stages of disease, suggesting non-language influences on some aspects of performance. Subsequent examination of these measures revealed significant linear trends over the three stages of disease in syntactic complexity, semantic and lexical content. The findings suggest, first, that there is a progressive disruption in language integrity, detectable from the prodromal stage in a subset of patients with Alzheimer’s disease, and secondly that measures of semantic and lexical content and syntactic complexity best capture the global progression of linguistic impairment through the successive clinical stages of disease. The identification of disease-specific language impairment in prodromal Alzheimer’s disease could enhance clinicians’ ability to distinguish probable Alzheimer’s disease from changes attributable to ageing, while longitudinal assessment could provide a simple approach to disease monitoring in therapeutic trials.

## Introduction

One of the most important clinical clues to a diagnosis of probable Alzheimer’s disease is an insidious history of learning and memory difficulties, often noticed by others, and sufficient to impact on performance of day-to-day activities (McKhann *et al.*, 1984; Dubois *et al.*, 2007). Detailed neuropsychological profiling of such patients, however, frequently demonstrates deficits across a range of other cognitive faculties, including higher visual processing (Locascio *et al.*, 1995; Rouleau *et al.*, 1996), frontal executive function (Lafleche and Albert, 1995; Bondi *et al.*, 2002), and language abilities (Locascio *et al.*, 1995; Garrard *et al.*, 2001). With progression of the disease, problems in all these additional domains become more prominent, leading to a typical end stage of global cognitive impairment (for review see Albert, 2011).

Much attention has been paid to the evolution of language change over the course of Alzheimer’s disease, as both retrospective analyses of written and spoken language dating from presymptomatic periods (Snowdon *et al.*, 1996; Garrard *et al.*, 2005; van Velzen and Garrard, 2008), and prospective cohort studies of ageing populations (Forbes-McKay and Venneri, 2005; Oulhaj *et al.*, 2009), have shown that subtle changes in language and communication ability may be apparent years or even decades before either a patient or his/her closest associates becomes aware of any symptoms of cognitive deterioration.

Translation of these striking observations into simple and specific markers of language change in Alzheimer’s disease could have far-reaching clinical consequences. The effortlessness of connected speech production in daily life makes it an easy biological sample to obtain, while the informational complexity of the data provides a multitude of analytical dimensions. The identification of disease-specific language abnormalities in patients with prodromal Alzheimer’s disease, or mild cognitive impairment (Petersen *et al.*, 1999; Gauthier *et al.*, 2006), would therefore enhance clinicians’ ability to distinguish probable Alzheimer’s disease from the more benign effects of ageing on cognition, while longitudinal assessment could provide readily obtainable markers of disease progression.

We recently examined connected speech samples obtained at the time of Alzheimer’s disease diagnosis from a series of patients in whom Alzheimer’s disease was subsequently confirmed at post-mortem. We found evidence of syntactic simplification (Ahmed *et al.*, 2012) and impairments in lexico-semantic processing (Ahmed *et al.*, 2013), in keeping with previous studies in clinically defined populations (Hier *et al.*, 1985; Croisile *et al.*, 1996; Giles *et al.*, 1996; Vuorinen *et al.*, 2000). The fact that these samples were drawn from a longitudinal study of ageing and dementia means that connected speech, along with other indices of language and cognition, can be serially assayed, capturing performance at both the mild cognitive impairment stage and later phases of disease progression in the same group of individuals. The objective of the present study was, therefore, to identify the features of connected speech that: (i) had been abnormal during the mild cognitive impairment phase of these patients’ illness; and (ii) showed consistently greater deviation from normal performance with disease progression.

## Materials and methods

### Participants

All participants had been recruited to the Oxford Project to Investigate Memory and Ageing (OPTIMA), a longitudinal study of the clinical, neuropsychological, biochemical and imaging correlates of ageing in community dwelling elderly persons with and without dementia [see Oulhaj *et al.* (2009) for fuller description of the OPTIMA design]. Ahmed *et al.* (2012) described the abnormalities present in the connected speech of 36 members of the OPTIMA cohort who had enrolled in the study between 1989 and 2006, either while cognitively healthy or with a diagnosis of mild cognitive impairment according to the Petersen *et al.* (1999, 2001) criteria. Eighteen members of this group had later progressed to meet criteria for probable Alzheimer’s disease, and the remaining participants continued to display normal cognition. All participants had been followed serially at 6 to 12 month intervals until death, and all brains submitted for post-mortem histological examination. Diagnoses of definite Alzheimer’s disease [according to the Consortium to Establish a Registry for Alzheimer’s Disease (CERAD) criteria (Mirra *et al.*, 1991)], with Braak stage V/VI neurofibrillary tangle density (Braak and Braak, 1991), had been found in all progressors, whereas minimal or no Alzheimer’s disease pathology had been present in control brain tissue.

For the present study we selected, from the same group of participants, all those for whom clinical and neuropsychological evaluation had supported a diagnosis of mild cognitive impairment between 6 and 18 months before the first assessment that had led to a classification of probable Alzheimer’s disease. Data at the mild cognitive impairment stage were unavailable in 3 of 18 patients. Cross-sectional data from 15 healthy elderly control participants, age and education matched and with no significant difference in gender ratio, were consecutively selected from the OPTIMA database as a comparison group. The absence of Alzheimer’s disease changes from all control brains was confirmed at post-mortem. The demographic characteristics of the patient subgroups and the matched control samples are displayed in Table 1.

**Table 1:** Demographic characteristics and neurospsychological scores for healthy controls and patients with Alzheimer’s disease at mild cognitive impairment and mild stages

	Healthy control subjects		Mild cognitive impairment^a^		Mild Alzheimer’s disease	
	(*n* = 15)		(*n* = 15)			
	Mean	SD	Mean	SD	Mean	SD
*Demographics*						
Age (years)	76.0	5.6	71.2	8.5	71.8	7.9
Education (years)	14.3	3.6	13.1	3.0		
Gender (male:female)	8:7		9:6			
MMSE (30)	29.1	1.0	24.7****	3.6	21.9****	3.2
*CAMCOG scores*^†^						
Total (107)	101.0	1.9	83.7****	5.7	77.7****	7.6
Orientation (10)	9.9	0.35	8.2**	2.0	7.1***	2.4
Comprehension (9)	8.9	0.26	8.4	1.0	8.0****	0.76
Expression (21)	19.5	1.2	16.9****	1.6	16.3****	2.0
Remote memory (6)	5.8	0.41	4.6**	1.2	4.3**	1.5
Recent memory (4)	3.9	0.26	2.9****	0.86	2.7***	1.1
Learning memory (17)	14.4	1.2	7.9****	3.6	7.7****	3.7
Attention (7)	6.7	0.59	5.6**	1.4	4.9**	2.0
Praxis (12)	11.7	0.46	11.1*	1.1	9.8****	1.4
Calculation (2)	2.0	0	1.7	0.73	1.7	0.59
Abstract thinking (8)	7.6	0.91	6.6*	1.5	5.7**	2.0
Perception (11)	10.5	0.64	10.0	1.2	9.5**	1.0

Maximum scores given in parentheses. Comparisons between controls and patient groups computed using *t*-tests and Chi-Square for comparison of gender ratio; **P < 0.01, ***P < 0.001, ****P < 0.0001. SD = standard deviation; MMSE = Mini-Mental State Examination.

† *n* = 14 for all MCI CAMCOG scores. Data were missing for one patient.

a Mean time to mild Alzheimer’s disease = 10.7 months; range 6–18 months.

To examine language performance at later disease stages, we further selected those participants for whom neuropsychological data were available both at the point of conversion to Alzheimer’s disease, and after disease severity had progressed from the mild (Mini-Mental State Examination 21–24) to moderate (Mini-Mental State Examination 10–20) stage. Data were available in nine patients, for whom nine age and education matched healthy elderly controls were selected as a comparison group. The demographic characteristics of patients at three clinical stages of Alzheimer’s disease (mild cognitive impairment, mild and moderate stage) and the matched control samples are displayed in Table 2. Detailed information on selection criteria for each phase of the study can be found in Supplementary Fig. 1.

**Table 2:** Demographic characteristics and neurospsychological scores for healthy control subjects and patients with Alzheimer’s disease at three clinical stages

	Healthy control subjets		Mild cognitive impairment		Mild Alzheimer’s disease^a^		Moderate Alzheimer’s disease^b^	
	(*n* = 9)		(*n* = 9)					
	Mean	SD	Mean	SD	Mean	SD	Mean	SD
*Demographics*								
Age (years)	75.6	5.4	72.0	6.5	73.3	6.1	75.1	6.2
Education (years)	13.8	3.6	12.8	3.2				
Gender (male:female)	4:5		6:3					
MMSE (30)	29.2	0.97	24.2*	4.5	22.2****	2.7	12.9****	6.3
*CAMCOG scores*								
Total (107)	100.9	2.4	83.2****	6.9	75.7****	6.2	48.2****	23.8
Orientation (10)	9.8	0.44	7.9*	2.3	6.7**	2.7	2.9****	2.7
Comprehension (9)	8.9	0.33	8.6	0.88	8.1*	0.78	6.3**	2.4
Expression (21)	19.6	1.6	16.9**	1.8	16.1***	2.0	11.0**	6.0
Remote memory (6)	5.9	0.33	4.7*	1.2	4.2*	1.6	2.4***	2.2
Recent memory (4)	3.9	0.33	2.8**	0.83	2.7*	1.2	1.1****	1.4
Learning memory (17)	14.9	1.1	6.6****	3.8	6.0****	3.8	3.1****	2.4
Attention (7)	6.7	0.71	5.9	1.5	5.6	1.7	2.1****	2.5
Praxis (12)	11.7	0.5	10.9	1.2	9.3****	1.2	6.9**	3.8
Calculation (2)	2.0	0	1.8	0.67	1.7	0.71	1.0**	0.87
Abstract thinking (8)	7.3	1.1	7.0	1.3	5.8	2.1	5.1	2.9
Perception (11)	10.3	0.71	10.3	0.87	9.6	0.88	6.2**	3.3

Maximum scores given in parentheses. Comparisons between controls and patient groups computed using *t*-tests and Chi-Square for comparison of gender ratio; *P < 0.05; **P < 0.01, ***P < 0.001, ****P < 0.0001.

a Mean time to mild Alzheimer’s disease = 10.0 months; range 6–18 months.

b Mean time to moderate Alzheimer’s disease = 24.0 months; range 19–30 months.

### Cognitive and linguistic assessment

All OPTIMA participants were evaluated using the CAMDEX interview (Roth *et al.*, 1986), which incorporates the CAMCOG, a brief neuropsychological battery focusing on cognitive abilities important to a diagnosis of dementia, namely: orientation, comprehension, expression, recent memory, remote memory, learning, abstract thinking, perception, praxis, attention and calculation. The Mini-Mental State Examination score was also extracted from the CAMCOG for all participants.

The language component of the CAMCOG includes elicitation of a sample of connected speech using the Cookie Theft picture description task from the Boston Diagnostic Aphasia Examination (Goodglass and Kaplan, 1983). As all assessments were routinely tape recorded, speech samples were available for transcription and further analysis.

Cookie Theft descriptions were transcribed following the conventions described by Garrard *et al.* (2011), and analysed using the method described by Wilson *et al.* (2010), with minor adaptations as documented in Ahmed *et al.* (2012). This approach uses the classification of normal and abnormal discourse proposed by Saffran *et al.* (1989), and uses the quantitative production analysis techniques described by Berndt *et al.* (2000). Briefly, the variables analysed were grouped under four headings: (i) speech production (speech rate, distortions, and phonological paraphasias); (ii) syntactic complexity (mean length of utterance, proportion of words in sentences, number of embedded clauses, syntactic errors, nouns preceded by determiners and verbs with inflections); (iii) lexical content [proportional frequencies of open class (nouns, verbs and descriptive terms) and closed class (grammatical function) words]; and (iv) fluency errors (false starts, repaired sequences, filled pauses and incomplete sentences).

Semantic content of the samples was quantified separately, using the semantic units classification described by Croisile *et al.* (1996), in which 23 units, relating to the four components of the picture, are assumed to constitute a complete description of the pictured scene: three subjects (boy, girl and mother), two locations (kitchen and exterior seen through the window), 11 objects (cookie, jar, stool, sink, plate, dishcloth, water, window, cupboard, dishes and curtains), and seven actions or attitudes (boy taking or stealing, boy or stool falling, woman drying or washing dishes/plate, water overflowing or spilling, action performed by the girl, woman unconcerned by the overflowing, woman indifferent to the children). Two additional measures—idea density (defined as the total number of semantic units divided by total number of words in a speech sample) and efficiency (the total number of semantic units divided by duration of the speech sample in seconds)—were also computed (Ahmed *et al.*, 2013).

All analyses were restricted to utterances connected with the stimulus picture, and ignored unrelated comments such as questions about the task or conversations with the examiner. Transcripts were analysed by two raters, resulting in good initial inter-rater agreement, and later consensus on all points of discrepancy.

### Statistical analysis

Linguistic variable scores were converted to z-scores using the control means and standard deviations. For those measures (i.e. error rates or counts of individual lexical items) on which higher raw scores were associated with greater cognitive impairment, the sign of the z-scores were reversed, allowing all figures and corresponding tables to be read as lower scores indicating more impairment. Independent samples *t*-tests were used to compare demographic and cognitive measures between controls and patients. Mann-Whitney U-tests were used to compare linguistic measures between controls and patients, and Wilcoxon signed-rank tests to compare linguistic measures between the clinical stages of Alzheimer’s disease (i.e. mild cognitive impairment, mild and moderate stage Alzheimer’s disease). Bonferroni correction for multiple comparisons was applied to all *P*-values. Finally, a repeated measures ANOVA was used to investigate linear trends in the quantitative production analysis variables, followed by paired sample *t*-tests to investigate differences between clinical stages.

## Results

### Connected speech abnormalities at the mild cognitive impairment stage

Performance on the Mini-Mental State Examination and all subtests of the CAMCOG other than those relating to comprehension, perception and calculation were significantly lower in the mild cognitive impairment group than those of control subjects, but the mean score was still above the dementia cut-off of 80/107 points. In contrast, and in agreement with the findings in the larger Alzheimer’s disease cohort reported by Ahmed *et al.* (2012), only scores on the calculation subtest of the CAMCOG were comparable with control performance when participants had reached the stage of mild Alzheimer’s disease (Table 1).

**Figure 1:**
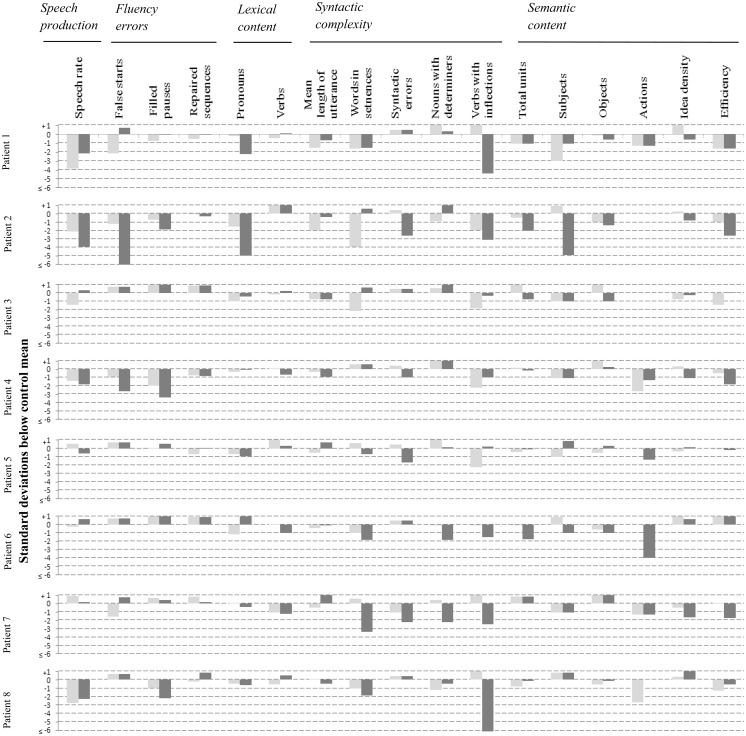

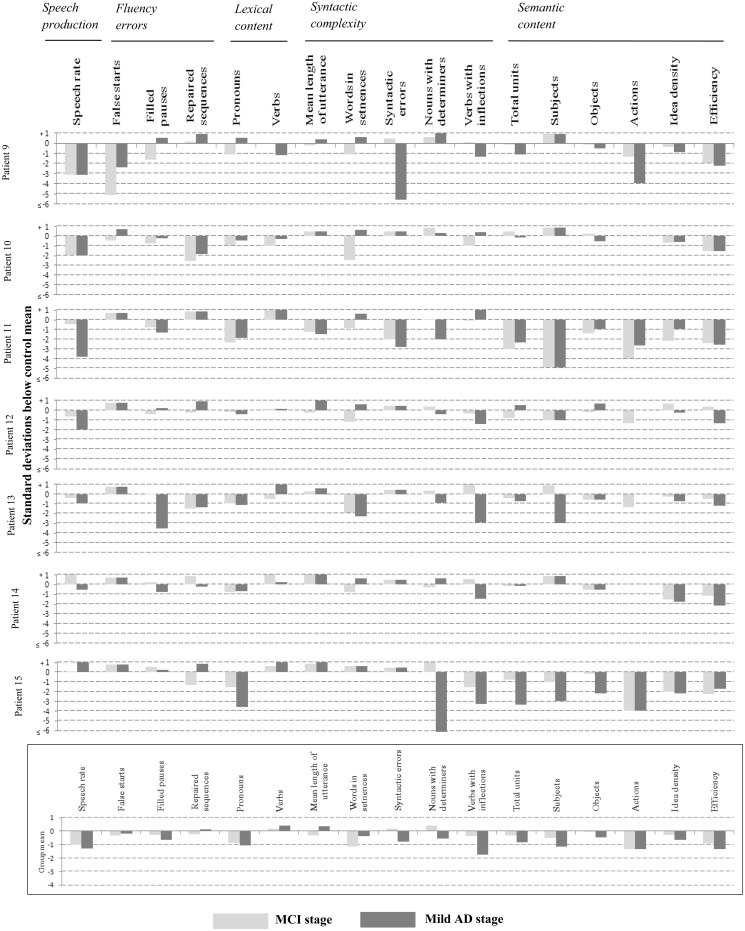
Individual case analysis of language profiles in patients, at mild cognitive impairment and mild Alzheimer’s disease stages. Each linguistic variable is associated with two bars. The light grey bar corresponds to performance at the mild cognitive impairment stage, and the dark grey bar corresponds to performance at the mild Alzheimer’s disease (AD) stage. Numerical values of all z-scores are provided in Supplementary Table 1.

Figure 1 displays (in the form of z-scores) all the quantitative production analysis and semantic content measures on which at least one transcript was associated with a score that fell 1.5 or more standard deviations below the control mean. Each speech variable is associated with two bars, the light grey bar representing performance at the mild cognitive impairment stage, and the dark grey bar performance at the mild Alzheimer’s disease stage.

Figure 1 shows that deficits were found in all mild cognitive impairment transcripts: 11 of 15 patients showed impairment in syntactic complexity and semantic content that was also observed as disease progressed to the early Alzheimer’s disease stage; six showed impairments in speech production and four in fluency errors, whereas only two patients showed changes in lexical content. However, Fig. 1 also indicates that a small proportion (8.2%) of the abnormalities associated with the mild cognitive impairment transcripts had become attenuated (i.e. improved but still within the impaired range), and 20% had disappeared entirely by the time the same patient met criteria for Alzheimer’s disease. Statistical comparison of group performance (Fig. 1) between mild cognitive impairment and mild Alzheimer’s disease stage on each of the individual linguistic indices, showed no significant differences after correction for multiple comparisons, though group differences in mean length of utterance (*P = *0.01) and syntactic errors (*P = *0.03) approached significance.

### Language markers of Alzheimer’s disease progression

In the patient subgroup for whom language samples were available for transcription and analysis at the moderate Alzheimer’s disease stage, and their matched controls, patients performed at significantly lower levels at all clinical stages on the Mini-Mental State Examination, CAMCOG total, orientation, expression, remote and recent memory (Table 2). Patients were significantly impaired on comprehension and praxis at the mild and moderate stages, and on attention, calculation and perception at the moderate stage only. There was no significant difference between controls and patients at any clinical stage on abstract thinking.

To identify the language variables that were likely to be useful as markers of cognitive change with clinical progression, we considered all variables on which the majority of cases showed a consistent decline across the stages of mild cognitive impairment and early Alzheimer’s disease (that is to say, cases in which impairment at the mild Alzheimer’s disease stage either appeared *de novo* or was more profound than that seen at the mild cognitive impairment stage). The variables remaining (and the linguistic domains to which they belonged) after this criterion was applied were: speech rate (speech production); filled pauses (fluency errors); proportions of pronouns and verbs (lexical content); proportion of words in sentences, syntactic errors, proportions of nouns with determiners and verbs with inflections (syntactic complexity); total semantic units and references to objects, subjects and actions, efficiency and idea density (semantic processing). These measures were used to examine the longitudinal profiles of the nine patients whose connected speech at the moderate dementia stage was available for characterization.

**Figure 2:**
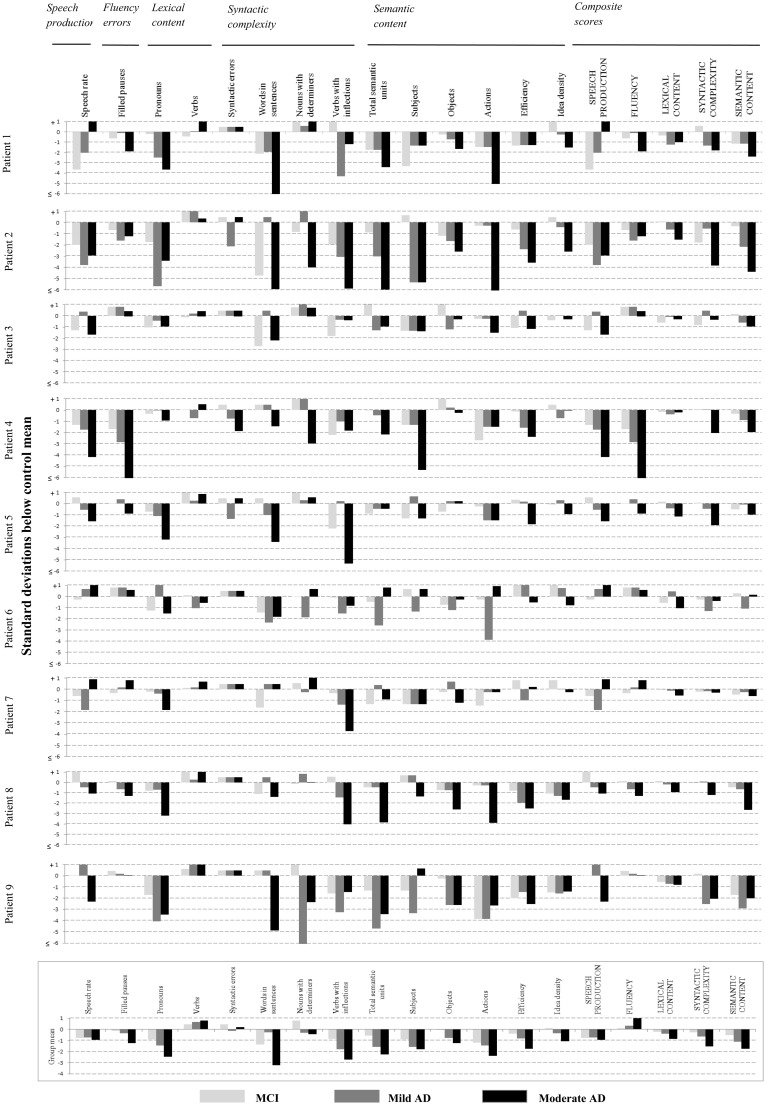
Individual case analysis of selected stable language variables, in patients with Alzheimer’s disease (AD) at three clinical stages. Each linguistic variable is associated with three bars, corresponding to performance at the mild cognitive impairment (MCI) stage (light grey), mild Alzheimer’s disease stage (dark grey), and moderate Alzheimer’s disease stage (black). Numerical values of all z-scores are provided in Supplementary Table 2.

Figure 2 shows the individual and group average language profiles of these patients across all three clinical stages. An increase in the production of pronouns, decrease in total semantic units and decrease in efficiency, showed a pattern of increasing impairment from the mild cognitive impairment stage through disease in over half of the patients, with the remaining patients showing impairments that were more variable. Consistent reductions in the identification of objects and actions were also noted in the majority of patients, but beginning later in the disease at the mild Alzheimer’s disease stage.

Statistical comparisons of group performance (Fig. 2) between each clinical stage showed no significant differences after correction for multiple comparisons (expected with the small numbers involved). However, without application of stringent corrections, there were a number of comparisons between mild cognitive impairment and moderate stage Alzheimer’s disease that were significant. These differences were evident in the proportion of pronouns (*P = *0.01), total semantic units (*P = *0.05), references to objects (*P = *0.02), idea density (*P = *0.02) and efficiency (*P = *0.02).

To define more robust measures of progression, we grouped the variables by linguistic domain, and computed a composite score of language performance specific to each domain. Language composite scores were derived by first computing z-scores as a function of the control mean, for each variable, and then averaging the z-scores of the constituent variables under each linguistic domain. These values were used to explore the possibility that composite measures might show a more consistent decline through the disease stages.

The results of composite analysis are shown in the right columns of Fig. 2. Although inconsistencies remained, they were fewer, so that the trajectory of decline mirrored disease stage in the majority of cases. Semantic content and syntactic complexity were the most frequently observed deficits, and decline on both these measures could be detected at the mild cognitive impairment stage.

**Figure 3:**
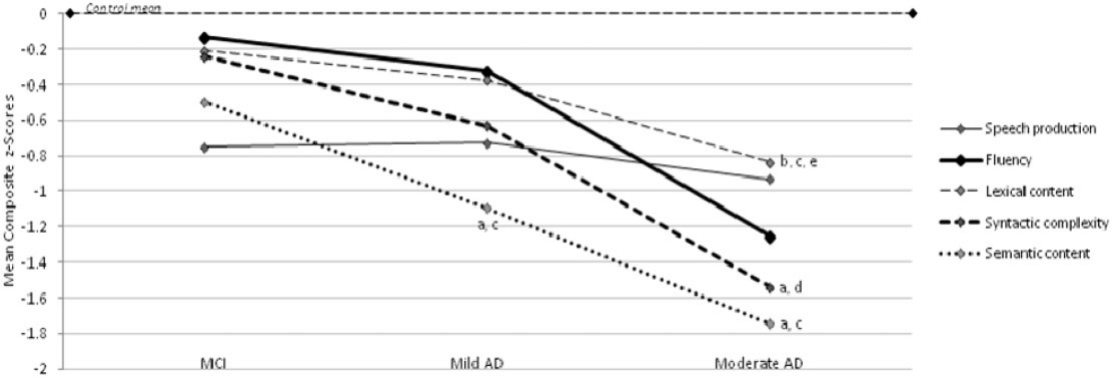
Linear trends in language composite scores across three clinical stages of Alzheimer’s disease (AD). ^a^Significant difference compared with control subjects, *P < *0.01. ^b^Significant difference compared with control subjects *P < *0.001. ^c^Significant difference compared with mild cognitive impairment (MCI) stage, *P < *0.05. ^d^Significant difference compared with mild cognitive impairment stage, *P < *0.01. ^e^Significant difference compared with mild Alzheimer’s disease, *P < *0.05.

We then looked for statistically significant trends in the decline of language performance. Because of the small sample size and the large number of individual variables, only composite scores were considered, in order to minimize reporting of false positive trends. A repeated measures ANOVA was used to examine language performance of the nine cases with Alzheimer’s disease at three clinical stages of disease, using severity as the within-subjects factor, and semantic content, syntactic complexity, speech production, lexical content and fluency error composite measures as dependent variables. On these measures, there were significant linear trends over the mean values for syntactic complexity [*F*(1,8) = 12.304, *P < *0.01], semantic content [*F*(1,8) = 8.627, *P < *0.05], and lexical content [*F*(1,8) = 9.084, *P < *0.05], but not for speech production [*F*(1,8) = 0.031, *P > *0.05] or fluency errors [*F*(1,8) = 2.735, *P > *0.05]. That the significant trends are in the direction of consistently greater impairment at successive clinical stages can be appreciated by inspection of Fig. 3. Pairwise comparisons showed that between controls and mild Alzheimer’s disease performance, there was a significant difference in semantic content (*t* = 3.095, *P < *0.01). Between controls and moderate stage performance, there was a significant difference in lexical content (*t* = 4.114, *P < *0.001), syntactic complexity (*t* = 3.768, *P < *0.01) and semantic content (*t* = 3.617, *P < *0.01), and significant differences in these variables were also seen between mild cognitive impairment and moderate Alzheimer’s disease performance (lexical content, *t* = 3.014, *P < *0.05; syntactic complexity, *t* = 3.508, *P < *0.01; semantic content, *t* = 2.937, *P < *0.05). Further significant differences between clinical stages were evident between mild cognitive impairment and mild Alzheimer’s disease in semantic content (*t* = 2.326, *P < *0.05) and between mild and moderate Alzheimer’s disease in lexical content (*t* = 2.432, *P < *0.05).

In addition, a number of differences between groups approached significance: controls versus mild cognitive impairment in semantic content (*t* = 1.820, *P = *0.088); controls versus mild Alzheimer’s disease in syntactic complexity (*t* = 1.813, P = 0.089) and lexical content (*t* = 1.772, *P = *0.095); and mild versus moderate Alzheimer’s disease in syntactic complexity (*t* = 2.096, *P = *0.069).

## Discussion

Few longitudinal studies have monitored language performance from mild cognitive impairment through to later stages of the disease. Tomoeda and Bayles (1993) followed three patients with Alzheimer’s disease longitudinally and, using the Cookie Theft description task, found that a reduction in conciseness was the best method for differentiating patients from controls, and the reduction of information units was the best measure of decline over time. In a larger longitudinal study, Tomoeda *et al.* (1996) examined five mild cognitive impairment patients and a larger group of patients with Alzheimer’s disease, confirming previous findings, and additionally reporting an increase in ideational repetitions. Clinical confirmation of conversion to Alzheimer’s disease was, however, not available for patients with mild cognitive impairment in either study. In light of the variable conversion rate in this group, such information is critical.

The present study documented the changes that took place in spoken discourse over the course of three well-defined clinical stages in nine patients, and is therefore not only the largest, but the first of its kind to have been conducted in patients with later neuropathological confirmation of the presence of the disease. First, the findings suggested that subtle changes in spoken language may be evident during prodromal stages of Alzheimer’s disease; and second that it was possible to derive measures of language function whose changing values mirrored global progression through the successive clinical stages of disease.

We began by adopting an individual case analysis, since reliance on group comparisons would have overlooked the spectrum of individual profiles of impairment. The results showed that there were significant changes in language in two-thirds of the group, an average of 12 months before clinical diagnosis of Alzheimer’s disease. It was also clear, however, that the abnormalities found were heterogeneous rather than conforming to a common profile. The latter is consistent with the heterogeneity typically observed in clinically probable Alzheimer’s disease in contrast to the more stereotyped patterns of language abnormality that are associated with the syndromes of primary progressive aphasia (Gorno-Tempini *et al.*, 2011).

Scrutiny of the pattern of evolution of these deficits at two later disease stages—newly diagnosed Alzheimer’s disease and moderate Alzheimer’s disease—suggested that abnormal performance on some aspect of discourse production at an earlier disease stage did not guarantee abnormality at later stages, implying that at least some of the measures were sensitive not only to deteriorating cognition but also to attentional, motivational, affective or other unstable influences on cognitive performance [though cognitive fluctuation has also been described in the context of Alzheimer’s disease (Hodges *et al.*, 2006)]. There is also evidence that casts doubt on the test–retest reliability of smaller samples of connected speech: Brookshire and Nicholas (1994) examined this property of three linguistic measures (words per minute, correct information per min, and per cent correct information units), in both small (<100 words) and larger samples of connected speech, and argued that these measures did not produce reliably similar sets of values when smaller samples were examined. Speech rate corresponded to words per minute studied by Brookshire and Nicholas (1994), and the instability of this measure across multiple testing episodes was confirmed by our results. The other of Brookshire and Nicholas’s (1994) measures (correct information per minute and per cent correct information units) mapped to our efficiency and total semantic unit measures, and these were among the indices that showed a largely consistent increase in impairment at successive disease stages, though both remained within the normal range throughout the period of follow-up in four and three patients, respectively, of the nine studied.

In general, the majority of individual measures that could be quantified using quantitative production analysis either differed from normal controls in only a small number of cases (e.g. the proportion of verbs used), or were subject to marked fluctuation over successive test sessions (e.g. the number of words in sentences), or both (e.g. the rate of filled pauses). In an attempt to detect a signal amid this obvious noise, we derived a series of composite scores that reflected performance within each of the domains of assessment covered by quantitative production analysis, together with a semantic composite. Of these, we found that the semantic, syntactic complexity and lexical content composites, but not speech production or fluency, showed statistically significant changes with disease progression. The overall direction of change in these variables was one of consistent deterioration with increasing disease. Furthermore, significant differences on these variables were found between controls and the mild Alzheimer’s disease stage, and importantly between mild cognitive impairment and moderate stages of disease, confirming the significant decline in these linguistic functions.

A decline in semantic content is consistent with previous findings from our group (Ahmed *et al.*, 2013) and others (Nicholas *et al.*, 1985), that connected spoken discourse in early Alzheimer’s disease is characterized by ‘empty’ speech, containing a high proportion of words and utterances that communicate little or no information. The accompanying decline in syntactic complexity was in line with the findings of our recent cross-sectional study of connected speech in mild Alzheimer’s disease (Ahmed *et al.*, 2012). The change in lexical content was largely driven by an increase in the use of pronouns, a finding that has been reported by others (Ripich and Terrell, 1988; Almor *et al.*, 1999), and attributed by one study to an underlying impairment of working memory (Almor *et al.*, 1999).

There were no significant changes in fluency or in speech production (in particular phonological errors and distortion of speech) over the course of disease. We have previously asked the question of whether the syndrome of isolated, progressive logopenic aphasia, in which Alzheimer’s disease has been found to be the most commonly underlying pathological process (Gorno-Tempini *et al.*, 2008; Rohrer *et al.*, 2012), may be a clinical feature of typical Alzheimer’s disease (Ahmed *et al.*, 2012). Logopenic aphasia is characterized by slow production rate and sparse phonological paraphasias (Gorno-Tempini *et al.*, 2008, 2011). Even at 2 years post-diagnosis, these core clinical features of logopenic aphasia remained largely absent in typical Alzheimer’s disease, lending further support to our earlier conclusion that logopenic aphasia is a clinical variant, rather than a clinical feature, of Alzheimer’s disease.

In conclusion, we suggest that in a subset of patients with Alzheimer’s disease there is progressive disruption in language integrity, detectable from prodromal stages, which is best captured by measures of semantic and lexical content and syntactic complexity. The circumstances under which the data were acquired allowed the stages of disease evolution to be accurately defined, and the availability of pathological diagnoses left no doubt about the nature of the degenerative lesion. However, the small volumes of language sampled and the susceptibility of such samples to variation across test sessions means that more intensive language sampling in a larger, independent cohort of patients is required in order to validate these findings. Correlating such language changes with anatomical differences on MRI [as Wilson *et al.* (2010) did in primary progressive aphasia], would add a valuable neuroanatomical dimension to the data. When these additional studies are accomplished, we believe that they will have at least three important clinical implications for the study of language change in Alzheimer’s disease and other neurodegenerative dementias. First, an improved understanding of language deficits in Alzheimer’s disease would guide the development of methods for improving communication between patients and their caregivers. Second, comparing deficits in specific aspects of connected speech in Alzheimer’s disease would contribute to the development of biologically important ‘deep phenotypes’ of the condition. Finally, language monitoring could represent a simple, rapid and reproducible approach to disease monitoring in therapeutic trials aimed at slowing progression and cognitive deterioration.

## Supplementary Material

Supplementary Data
