# Supplementary material for: Connected speech as a marker of disease progression in autopsy-proven Alzheimer’s disease
Source: Brain. 2013 Oct 18;136(12):3727–37. doi: 10.1093/brain/awt269 (PMC3859216; doi:10.1093/brain/awt269)
Supplement: Supplementary Data [file supp_136_12_3727__index.html]

Connected speech as a marker of disease progression in autopsy-proven Alzheimer’s disease — Supplementary Data 

# Connected speech as a marker of disease progression in autopsy-proven Alzheimer’s disease

## Supplementary Data

files

**Files in this Data Supplement:**

- Supplementary Data - doc file
- Supplementary Data - doc file
- Supplementary Data - doc file
