# Supplementary material for: Connected speech as a marker of disease progression in autopsy-proven Alzheimer’s disease
Source: Brain. 2013 Oct 18;136(12):3727–37. doi: 10.1093/brain/awt269 (PMC3859216; doi:10.1093/brain/awt269)
Supplement: Supplementary Data [file supp_awt269_brain-2013-00704-File007.doc]

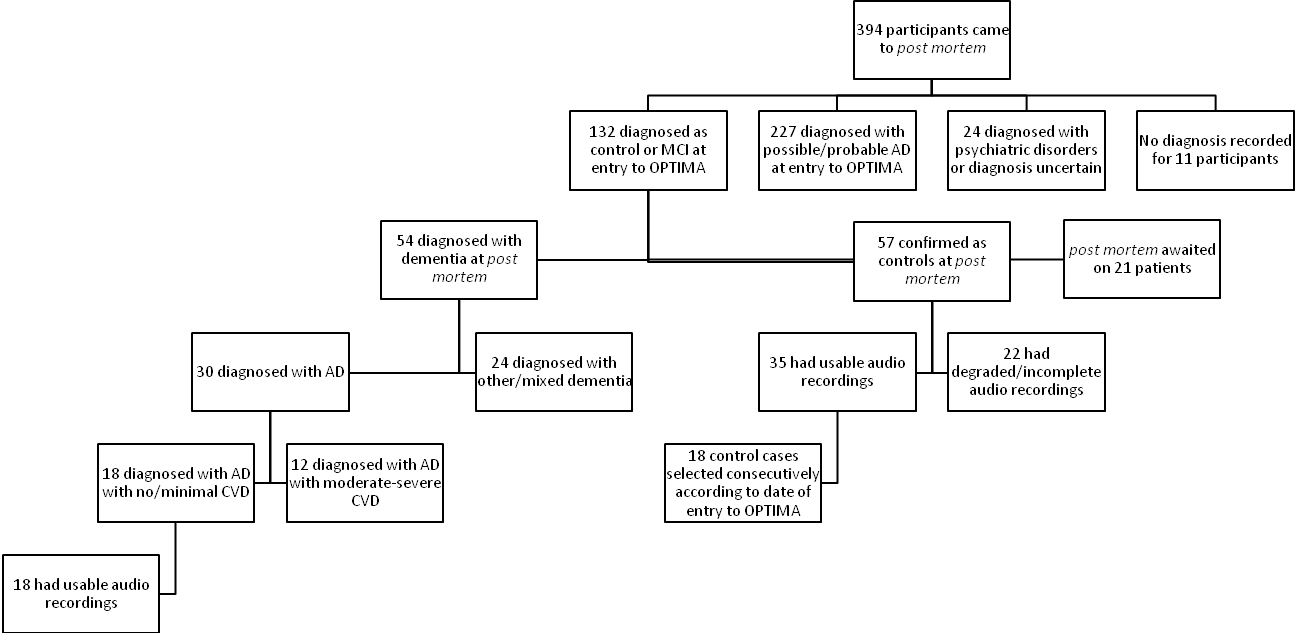

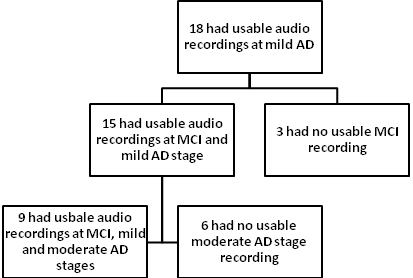


*Study sample for analysis of connected speech in MCI*

*Study sample for language markers of disease progression*

*Healthy control sample*

*Study sample for Ahmed et al. JNNP 2012; 83:1056-1062.*

**Supplementary Figure 1.** Flowchart to illustrate subject attrition due to selection criteria. Study samples used in a previous study (Ahmed et al. 2012) and the current study are highlighted.
