# Supplementary material for: Connected speech as a marker of disease progression in autopsy-proven Alzheimer’s disease
Source: Brain. 2013 Oct 18;136(12):3727–37. doi: 10.1093/brain/awt269 (PMC3859216; doi:10.1093/brain/awt269)
Supplement: Supplementary Data [file supp_awt269_brain-2013-00704-File008.doc]

|  |  | SPEECH PRODUCTION | FLUENCY ERRORS | | | LEXICAL CONTENT | | SYNTACTIC COMPLEXITY | | | | | SEMANTIC CONTENT | | | | | |
| --- | --- | --- | --- | --- | --- | --- | --- | --- | --- | --- | --- | --- | --- | --- | --- | --- | --- | --- |
| Clinical stage | Case no. | Speech rate | False starts | Filled pauses | Repaired sequences | Pronouns | Verbs | Mean length of utterance | Words in sentences | Syntactic errors | Nouns with determiners | Verbs with inflections | Total units | Subjects | Objects | Actions | Idea density | Efficiency |
|  |  |  |  |  |  |  |  |  |  |  |  |  |  |  |  |  |  |  |
| MCI | AD1 | -3.82 | -2.16 | -0.74 | -0.55 | -0.20 | -0.43 | -1.52 | -1.63 | 0.46 | 1.70 | 2.00 | -1.08 | -2.97 | -0.14 | -1.32 | 1.23 | -1.61 |
| MCI | AD2 | -2.08 | -1.20 | -0.74 | -0.07 | -1.55 | 1.87 | -1.92 | -3.89 | 0.46 | -0.90 | -1.97 | -0.45 | 0.90 | -0.97 | 0 | 0.31 | -0.92 |
| MCI | AD3 | -1.38 | 0.73 | 1.01 | 0.88 | -0.96 | -0.14 | -0.77 | -2.12 | 0.46 | 0.54 | -1.85 | 1.79 | -1.03 | 1.94 | 0 | -0.71 | -1.37 |
| MCI | AD4 | -1.39 | -0.93 | -2.01 | -0.77 | -0.33 | 0.07 | -0.36 | 0.63 | 0.46 | 1.31 | -2.23 | 0.19 | -1.03 | 1.52 | -2.65 | 0.34 | -0.45 |
| MCI | AD5 | 0.54 | 0.73 | 0.05 | -0.70 | -0.68 | 1.05 | -0.57 | 0.63 | 0.46 | 1.31 | -2.23 | -0.45 | -1.03 | -0.55 | 0 | -0.33 | -0.02 |
| MCI | AD6 | -0.30 | 0.73 | 1.01 | 0.88 | -1.15 | 0.09 | -0.42 | -1.02 | 0.46 | -0.11 | -0.11 | -0.13 | 0.90 | -0.55 | 0 | 1.00 | 0.95 |
| MCI | AD7 | 0.91 | -1.56 | 0.67 | 0.88 | 0.06 | -1.07 | -0.49 | 0.63 | -1.02 | 0.47 | 1.27 | 0.83 | -1.03 | 1.52 | -1.32 | -0.46 | 0.01 |
| MCI | AD8 | -2.74 | 0.73 | -0.94 | -0.19 | -0.41 | -0.55 | 0.07 | -1.05 | 0.46 | -1.23 | 0.99 | -0.77 | 0.90 | -0.55 | -2.65 | 0.40 | -1.28 |
| MCI | AD9 | -3.12 | -5.13 | -1.65 | 0.16 | -1.11 | 0 | -0.22 | -1.09 | 0.46 | 0.63 | 0.08 | -0.13 | 0.90 | -0.14 | -1.32 | -0.34 | -1.96 |
| MCI | AD10 | -1.91 | -0.41 | -0.72 | -2.52 | -0.91 | -1.03 | 0.51 | -4.29 | 0.46 | 0.84 | -1.02 | 0.51 | 0.90 | 0.28 | 0 | -0.64 | -1.56 |
| MCI | AD11 | -0.46 | 0.73 | -0.74 | 0.88 | -2.31 | 1.63 | -1.25 | -0.88 | -2.02 | 0.07 | -0.05 | -3.00 | -4.91 | -1.38 | -3.97 | -2.14 | -2.43 |
| MCI | AD12 | -0.65 | 0.73 | -0.40 | -0.28 | -0.20 | 0.12 | -0.29 | -1.20 | 0.46 | 0.36 | -0.35 | -0.77 | -1.03 | -0.14 | -1.32 | 0.72 | 0.40 |
| MCI | AD13 | -0.42 | 0.73 | 0.04 | -1.52 | -0.94 | -0.49 | 0.24 | -1.89 | 0.46 | 0.36 | 1.16 | -0.45 | 0.90 | -0.55 | -1.32 | -0.29 | -0.48 |
| MCI | AD14 | 1.80 | 0.73 | 0.22 | 0.88 | -0.74 | 1.04 | 1.10 | -0.74 | 0.46 | -0.27 | 0.54 | -0.13 | 0.90 | -0.55 | 0 | -1.55 | -1.11 |
| MCI | AD15 | 0.02 | 0.73 | 0.56 | -1.35 | -1.53 | 0.59 | 0.88 | 0.63 | 0.46 | 1.10 | -1.59 | -0.77 | -1.03 | -0.14 | -3.97 | -2.05 | -2.23 |
|  |  |  |  |  |  |  |  |  |  |  |  |  |  |  |  |  |  |  |
| Mild AD | AD1 | -2.12 | 0.73 | -0.08 | -0.01 | -2.24 | 0.06 | -0.67 | -1.48 | 0.46 | 0.36 | -4.35 | -1.08 | -1.03 | -0.55 | -1.32 | -0.57 | -1.56 |
| Mild AD | AD2 | -3.94 | -6.44 | -1.88 | -0.30 | -5.03 | 4.27 | -0.38 | 0.63 | -2.62 | 2.26 | -3.12 | -2.04 | -4.91 | -1.38 | 0 | -0.75 | -2.59 |
| Mild AD | AD3 | 0.36 | 0.73 | 1.01 | 0.88 | -0.41 | 0.21 | -0.74 | 0.63 | 0.46 | 1.02 | -0.35 | -0.77 | -1.03 | -0.97 | 0 | -0.22 | 0.08 |
| Mild AD | AD4 | -1.82 | -2.63 | -3.40 | -0.78 | -0.06 | -0.69 | -0.88 | 0.63 | -0.99 | 1.21 | -1.02 | -0.13 | -1.03 | 0.28 | -1.32 | -1.10 | -1.82 |
| Mild AD | AD5 | -0.60 | 0.73 | 0.51 | 0.06 | -0.98 | 0.27 | 0.67 | -0.67 | -1.69 | 0.15 | 0.24 | -0.13 | 0.90 | 0.28 | -1.32 | 0.12 | -0.18 |
| Mild AD | AD6 | 0.64 | 0.73 | 1.01 | 0.88 | 1.68 | -1.01 | -0.11 | -1.82 | 0.46 | -1.80 | -1.52 | -1.72 | -1.03 | -0.97 | -3.97 | 0.65 | 1.19 |
| Mild AD | AD7 | 0.17 | 0.73 | 0.39 | 0.21 | -0.43 | -1.23 | 1.11 | -3.37 | -2.18 | -2.19 | -2.45 | 0.83 | -1.03 | 1.52 | -1.32 | -1.62 | -1.73 |
| Mild AD | AD8 | -2.23 | 0.73 | -2.18 | 0.88 | -0.64 | 0.56 | -0.42 | -1.85 | 0.46 | -0.45 | -5.05 | -0.13 | 0.90 | -0.14 | 0 | 1.00 | -0.53 |
| Mild AD | AD9 | -3.14 | -2.41 | 0.54 | 0.88 | 0.55 | -1.20 | 0.38 | 0.63 | -5.60 | 1.00 | -1.34 | -1.08 | 0.90 | -0.55 | -3.97 | -0.92 | -2.25 |
| Mild AD | AD10 | -1.99 | 0.73 | -0.23 | -1.84 | -0.47 | -0.31 | 0.51 | 0.63 | 0.46 | 0.36 | 0.37 | -0.13 | 0.90 | -0.55 | 0 | -0.57 | -1.56 |
| Mild AD | AD11 | -3.78 | 0.73 | -1.31 | 0.88 | -1.84 | 1.20 | -1.50 | 0.63 | -2.83 | -2.05 | 1.22 | -2.36 | -4.91 | -0.97 | -2.65 | -0.92 | -2.59 |
| Mild AD | AD12 | -1.95 | 0.73 | 0.22 | 0.88 | -0.38 | 0.15 | 3.02 | 0.63 | 0.46 | -0.39 | -1.41 | 0.51 | -1.03 | 0.69 | 0 | -0.22 | -1.30 |
| Mild AD | AD13 | -1.00 | 0.73 | -3.54 | -1.35 | -1.15 | 1.0 | 0.58 | -2.31 | 0.46 | -0.90 | -2.88 | -0.77 | -2.97 | -0.55 | 0 | -0.75 | -1.21 |
| Mild AD | AD14 | -0.54 | 0.73 | -0.76 | -0.21 | -0.64 | 0.28 | 1.77 | 0.63 | 0.46 | 0.61 | -1.46 | -0.13 | 0.90 | -0.55 | 0 | -1.80 | -2.16 |
| Mild AD | AD15 | 2.93 | 0.73 | 0.24 | 0.88 | -3.60 | 2.51 | 2.23 | 0.63 | 0.46 | -7.22 | -3.29 | -3.32 | -2.97 | -2.21 | -3.97 | -2.15 | -1.73 |

**Supplementary Table 1.** Z-scores for each linguistic variable at MCI and mild AD stage (n=15). Scores rounded to two decimal places.
