# Supplementary material for: Connected speech as a marker of disease progression in autopsy-proven Alzheimer’s disease
Source: Brain. 2013 Oct 18;136(12):3727–37. doi: 10.1093/brain/awt269 (PMC3859216; doi:10.1093/brain/awt269)
Supplement: Supplementary Data [file supp_awt269_brain-2013-00704-File009.doc]

|  |  | SPEECH PRODUCTION | FLUENCY ERRORS | LEXICAL CONTENT | | SYNTACTIC COMPLEXITY | | | | SEMANTIC CONTENT | | | | | |
| --- | --- | --- | --- | --- | --- | --- | --- | --- | --- | --- | --- | --- | --- | --- | --- |
| Clinical stage | Case no. | Speech rate | Filled pauses | Pronouns | Verbs | Syntactic errors | Words in sentences | Nouns with determiners | Verbs with inflections | Total units | Subjects | Objects | Actions | Efficiency | Idea density |
| MCI | AD1 | -3.68 | -0.65 | -0.20 | -0.454 | 0.48 | -2.13 | 2.04 | 1.99 | -1.74 | -3.33 | -0.26 | -1.47 | -1.34 | 1.23 |
| MCI | AD2 | -1.99 | -0.65 | -1.73 | 1.92 | 0.48 | -4.73 | -0.84 | -1.95 | -0.89 | 0.67 | -1.20 | -0.27 | -0.61 | 0.47 |
| MCI | AD3 | -1.31 | 0.80 | -1.06 | -0.15 | 0.48 | -2.69 | 0.75 | -1.83 | 2.06 | -1.33 | 2.09 | -0.27 | -1.09 | -0.38 |
| MCI | AD4 | -1.32 | -1.71 | -0.35 | 0.07 | 0.48 | 0.48 | 1.61 | -2.21 | -0.05 | -1.33 | 1.62 | -2.67 | -0.11 | 0.49 |
| MCI | AD5 | 0.56 | 0 | -0.74 | 1.07 | 0.48 | 0.48 | 1.61 | -2.21 | -0.89 | -1.33 | -0.73 | -0.27 | 0.35 | -0.06 |
| MCI | AD6 | -0.26 | 0.80 | -1.27 | 0.09 | 0.48 | -1.43 | 0.04 | -0.11 | -0.47 | 0.67 | -0.73 | -0.27 | 1.39 | 1.04 |
| MCI | AD12 | -0.60 | -0.37 | -0.20 | 0.11 | 0.48 | -1.63 | 0.56 | -0.34 | -1.31 | -1.33 | -0.26 | -1.47 | 0.81 | 0.81 |
| MCI | AD14 | 1.78 | 0.14 | -0.81 | 1.07 | 0.48 | -1.11 | -0.14 | 0.54 | -0.47 | 0.667 | -0.73 | -0.27 | -0.81 | -1.08 |
| MCI | AD15 | 0.06 | 0.43 | -1.70 | 0.60 | 0.48 | 0.48 | 1.38 | -1.58 | -1.31 | -1.33 | -0.26 | -3.87 | -2.00 | -1.50 |
|  |  |  |  |  |  |  |  |  |  |  |  |  |  |  |  |
| Mild AD | AD1 | -2.03 | -0.10 | -2.51 | 0.05 | 0.48 | -1.96 | 0.56 | -4.31 | -1.74 | -1.33 | -0.73 | -1.47 | -1.29 | -0.27 |
| Mild AD | AD2 | -3.80 | -1.60 | -5.66 | 4.40 | -2.13 | 0.48 | 2.66 | -3.08 | -3.00 | -5.33 | -1.67 | -0.27 | -2.39 | -0.41 |
| Mild AD | AD3 | 0.38 | 0.80 | -0.44 | 0.21 | 0.48 | 0.48 | 1.29 | -0.34 | -1.31 | -1.33 | -1.20 | -0.27 | 0.46 | 0.02 |
| Mild AD | AD4 | -1.73 | -2.86 | -0.04 | -0.72 | -0.75 | 0.48 | 1.49 | -1.01 | -0.47 | -1.33 | 0.21 | -1.47 | -1.56 | -0.70 |
| Mild AD | AD5 | -0.55 | 0.39 | -1.08 | 0.27 | -1.34 | -1.02 | 0.33 | 0.24 | -0.47 | 0.667 | 0.21 | -1.47 | 0.18 | 0.31 |
| Mild AD | AD6 | 0.66 | 0.80 | 1.93 | -1.05 | 0.48 | -2.35 | -1.84 | -1.51 | -2.58 | -1.33 | -1.20 | -3.87 | 1.65 | 0.75 |
| Mild AD | AD12 | -1.87 | 0.15 | -0.40 | 0.15 | 0.48 | 0.48 | -0.28 | -1.40 | 0.38 | -1.33 | 0.68 | -0.27 | -1.01 | 0.02 |
| Mild AD | AD14 | -0.49 | -0.67 | -0.70 | 0.28 | 0.48 | 0.48 | 0.83 | -1.45 | -0.47 | 0.67 | -0.73 | -0.27 | -1.93 | -1.29 |
| Mild AD | AD15 | 2.89 | 0.16 | -4.04 | 2.59 | 0.48 | 0.48 | -7.84 | -3.26 | -4.69 | -3.33 | -2.61 | -3.87 | -1.47 | -1.58 |
|  |  |  |  |  |  |  |  |  |  |  |  |  |  |  |  |
| Moderate AD | AD1 | 3.19 | -1.89 | -3.65 | 1.66 | 0.48 | -8.57 | 2.04 | -1.19 | -3.42 | -1.33 | -1.67 | -5.07 | -1.29 | -1.53 |
| Moderate AD | AD2 | -2.93 | -1.24 | -3.40 | 0.36 | 0.48 | -5.94 | -4.02 | -5.88 | -5.96 | -5.33 | -2.61 | -6.27 | -3.58 | -2.60 |
| Moderate AD | AD3 | -1.64 | 0.44 | -0.91 | 0.41 | 0.48 | -2.16 | 0.72 | -0.34 | -0.89 | -1.33 | -0.26 | -1.47 | -1.13 | -0.27 |
| Moderate AD | AD4 | -4.17 | -7.86 | -0.91 | 0.51 | -1.88 | -1.46 | -2.99 | -1.83 | -2.16 | -5.33 | -0.26 | -1.47 | -2.37 | -0.03 |
| Moderate AD | AD5 | -1.57 | -0.88 | -3.19 | 0.86 | 0.48 | -3.39 | 0.56 | -5.36 | -0.47 | -1.33 | 0.21 | -1.47 | -1.83 | -0.95 |
| Moderate AD | AD6 | 1.18 | 0.59 | -1.52 | -0.60 | 0.48 | -1.82 | 0.66 | -0.85 | 0.80 | 0.67 | -0.26 | 0.93 | -0.52 | -0.79 |
| Moderate AD | AD12 | 0.90 | 0.80 | -1.86 | 0.69 | 0.48 | 0.48 | 1.49 | -3.74 | -0.89 | -1.33 | -1.20 | -0.27 | 0.22 | -0.27 |
| Moderate AD | AD14 | -1.05 | -1.29 | -3.19 | 1.30 | 0.48 | -1.40 | 0.04 | -4.01 | -3.85 | -1.33 | -2.61 | -3.87 | -2.49 | -1.67 |
| Moderate AD | AD15 | -2.31 | 0.06 | -3.45 | 1.85 | 0.48 | -4.87 | -2.36 | -1.46 | -3.42 | 0.67 | -2.61 | -2.67 | -2.52 | -1.42 |

**Supplementary Table 2.** Z-scores for each linguistic variable at MCI, mild and moderate AD stage (n=9). Scores rounded to two decimal places.
